# Individual mobility promotes punishment in evolutionary public goods games

**DOI:** 10.1038/s41598-017-12823-4

**Published:** 2017-10-25

**Authors:** Rui Cong, Qianchuan Zhao, Kun Li, Long Wang

**Affiliations:** 1 Center for Intelligent and Networked Systems, Department of Automation and TNList, Tsinghua University, Beijing, 100084 China; 2 Center for Systems and Control, College of Engineering, Peking University, Beijing, 100871 China

## Abstract

In explaining the pressing issue in biology and social sciences how cooperation emerges in a population of self-interested individuals, researchers recently pay intensive attentions to the role altruistic punishment plays. However, as higher-order cooperators, survival of punishers is puzzling due to their extra cost in regulating norm violators. Previous works have highlighted the importance of individual mobility in promoting cooperation. Yet its effect on punishers remains to be explored. In this work we incorporate this feature into modeling the behavior of punishers, who are endowed with a choice between leaving current place or staying and punishing defectors. Results indicate that optimal mobility level of punishers is closely related to the cost of punishing. For considerably large cost, there exists medium tendency of migration which favors the survival of punishers. This holds for both the direct competition between punishers and defectors and the case where cooperators are involved, and can also be observed when various types of punishers with different mobility tendencies fight against defectors simultaneously. For cheap punishment, mobility does not provide with punishers more advantage even when they are initially rare. We hope our work provide more insight into understanding the role individual mobility plays in promoting public cooperation.

## Introduction

Cooperation with non-kin is a fundamental and ubiquitous feature of human society^1–4^. A complicated dilemma derives from the fact that defectors gain an obvious advantage over cooperators. According to Darwin’s theory of evolution, competition rather than cooperation ought to drive our actions. Thus addressing the subtleties how cooperation emerges and stabilizes in groups continues to intrigue researchers in multidisciplinary fields^5–10^. Past decades have seen the paradigm of punishment rises as one of the more successful strategies by means of which cooperation might be promoted^11–18^. Indeed, our societies are home to a plethora of sanctioning institutions. However, punishment is costly, and as such it reduces the payoffs of both the defectors as well as of those that exercise the punishment. Punishers fall victim to the second-order free-riders, who cooperate but do not pay extra cost to punish defectors. As such survival of punishers in the evolution becomes puzzling. Up to now, a number of mechanisms that have been proved successful in explaining the evolution of cooperation are also available in explaining the evolution of punishment, such as the group selection^19^ and spatial structure^20–25^. Other mechanisms supporting punishment include the voluntary participation^26,27^, pool punishment^28^, coordinated punishment^29^, probabilistic punishment^30^. Yet the evolution of altruistic punishment remains an open problem.

Mobility is an essential feature of living organisms. Through migration, individuals free themselves from undesirable circumstances and negative consequences of that situation, in pursuit of more profitable environment^31^. Ample theoretical works have demonstrated the significant role migration plays in promoting or affecting the evolution of social cooperation^32–42^. In the framework of spatial games, by simply introducing a moderate probability of random migration to adjacent sites, cooperation level can be greatly enhanced^32,43,44^. Additional driving forces for migration as the payoff ^34,45^, aspiration^46^, as well as reputation^38^, can have diverse effects in elevating cooperation. However, as the second-order cooperator, punisher’s mobility is often ignored in previous studies. In fact, one of the key reasons that punishment can not establish in the population lies in its heavy cost when punishers are rare, who punish left and right in a sea of defectors and are quickly wiped out by natural selection. Previous works have shown that when punishment is probabilistic, the burden of punishing can be largely alleviated^30,47^. Nevertheless, when stuck in inferior environment, punishers do not necessarily need to execute this action aiming at modifying behavior (called partner control), but have an alternative choice of leaving^48–50^, to avoid future exploitation from such partners (called partner choice). The effect of such choice remains to be probed into.

Motivated by this, we here construct a spatial game model, using the typical paradigm of the public goods game (PGG) which exists in all kinds of complex social systems widely, such as the distribution of social welfare, environmental protection, public transportation, civil medical insurance and so on. Individuals are located on the sites of a network and play games with their direct neighbors indicated by the edges they are linked to. After the PGG which is organized in the group centered on each site, each punisher can decide to stay still and punish defectors or to leave but do not punish, judging by the number of defectors encountered in the game interaction. A threshold value is used here to govern the sensitivity of running away. Two scenarios are considered here: one in which punishers are directly faced with defectors, and the other where defectors and cooperators compete for survival in conflict against the same enemy, the defectors. By Monte Carlo simulations, we will show that the level of optimal punisher’s mobility is closely related to the cost of punishing. Unless for the negligible cost of punishing, there exists medium tendency of migration which favors the survival of punishers best. This conclusion holds for both the direct competition between punishers and defectors and the case where second-order free-riders are involved, and is also verified when various types of punishers with different mobility tendencies fight against defectors simultaneously.

This article argues for a closer look at the condition where punishers survive when the severeness of punishment can be diluted by alternative choice of running away in harsh situations. More precisely, we aim to identify the optimal conditions of migration tendency such that punishment can be sustained most at an equilibrium. The following is a summary of the rest of the paper. In section Methods we introduce the model. In section Results we present the main results. We make concluding remarks in the section Discussion.

## Results

**Figure 1:**
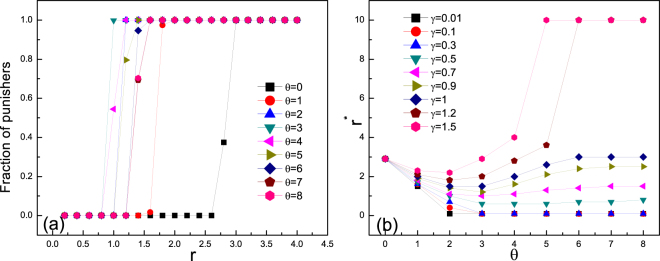
(**a**) Fraction of punishers as a function of enhancement factor $r$ for different values of threshold of migration $\theta$ with punishment cost $\gamma =0.7$ and intensity $\beta =1$. Notably, a medium value of $\theta$ favors punishers most under this circumstance. (**b**) Critical value of enhancement factor ${r}^{\ast }$ above which punishers dominate as a function of threshold of migration $\theta$ for different values of punishment cost $\gamma$ when $\beta =1$. Lower ${r}^{\ast }$ implies a more favorable environment for punishers. Note that for low punishment cost, to stay still and always to punish is a best choice. While for larger punishment cost, there exists medium value of $\theta$ that is optimal. Initially equal fractions 0.5 of punishers and defectors are randomly distributed among the population. Population density is $\rho =0.5$.

We start by considering the direct competition between punishers and defectors. Figure 1(a) shows the fraction of punishers as a function of enhancement factor $r$ for different values of threshold of migration $\theta$. Extreme case of $\theta =0$ implies that punishers “always run” as long as a defector exists in current group, while for the large value of $\theta$ equal to group size, punishers always stay still and punish. It can be seen that neither “always running” nor “always punishing” choice is most advantageous but a medium value ensures that punishers thrive at a lower enhancement factor $r$. Further investigation reveals that whether larger $\theta$ favors punishers relies on the cost of punishment. Critical value of enhancement factor ${r}^{\ast }$ above which punishers dominate as a function of threshold of migration $\theta$ for different values of punishment cost $\gamma$ is shown in Fig. 1(b). For low punishment cost, to stay still and always to punish is the best choice. While for larger punishment cost, there exists medium value of $\theta$ that is optimal. Note that always running ($\theta =0$) is never the best choice regardless of the cost, since the limit case of high mobility rates can be interpreted as a mixing favoring defection^33^, which can be well described by mean-field approximation^51,52^.

**Figure 2:**
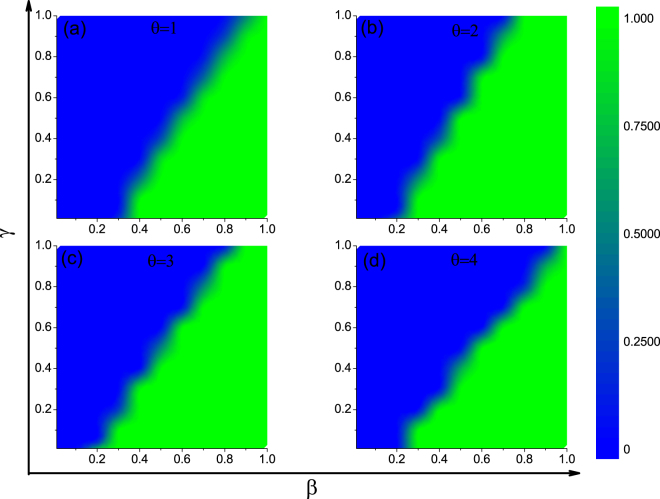
Fraction of punishers in dependence of punishing cost $\gamma$ and punishing intensity $\beta$ in the contour form when population are composed of D and P. Different panels correspond to different threshold to migrate $\theta =\mathrm{1,\; 2,\; 3,\; 4}$, respectively. The case for $\theta =0$ is not shown where defectors dominate for the whole given parameter plane. Initial fraction of P and D are both 0.5. Other parameters: $r=2$, and $\rho =0.5$.

A detailed revelation of how specific punishment parameters as both the cost $\gamma$ and intensity $\beta$ affect the evolution of punishers is demonstrated in Fig. 2 in a contour form. Sharp transitions between full defection and full punishment can be observed in the $\gamma$-$\beta$ plane. For any $\theta$, given a fixed value of cost, the intensity should be larger than a certain value for punishers to dominate; and conversely, for a given value of intensity (which should also be larger than a threshold), the cost should be smaller than a certain value for punishers to prevail. In general, the right bottom corner with high punishing intensity but low cost is most favorable for punishers. Note that this region is maximized neither by small $\theta$ nor but larger $\theta$ but medium one, indicating that a moderate migration tendency of punishers is most profitable. This also confirms the observation in Fig. 1, where punishment is effective with $\beta$ larger than a threshold.

**Figure 3:**
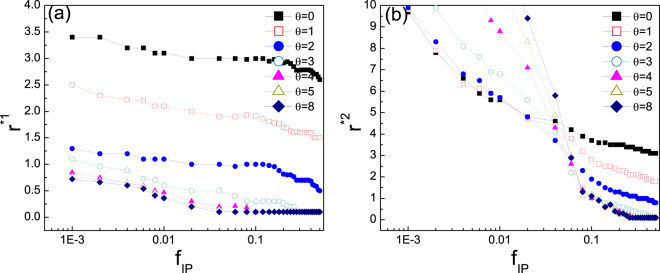
Critical value of $r$ as a function of initial fraction of punishers ${f}_{IP}$. (**a**) Critical value of enhancement factor ${r}^{\mathrm{\ast 1}}$, above which punishers emerge dependent on ${f}_{IP}$ for different values of $\theta$. (**b**) Critical value of enhancement factor ${r}^{\mathrm{\ast 2}}$, above which punishers dominate the population dependent on ${f}_{IP}$ for different values of $\theta$. The criterion for emergence and dominance of punishers are that the average stationary fractions reach 0.01 and 0.99, respectively. Other parameters: $\gamma =0.3$, $\beta =1$, and $\rho =0.5$.

Previous works have unveiled the fact that different initial distributions of a certain type of individuals in the whole population have significant impact on the final outcome of evolution in spatial games^53–56^. Hence it is necessary to probe into the different roles of punisher’s migration tendency under either adverse circumstances when relative rare or advantageous environment when sufficiently abundant. To this end, we have explored the evolution of punishers under different initial fractions from quite rare (0.001) to relatively abundant (0.5) in the population under cheap punishment (with relatively low cost compared to its effect, which is more commonly observed in reality). Figure 3 shows the critical value of enhancement factor ${r}^{\mathrm{\ast 1}}$ above which punishers emerge (in panel (a)) and ${r}^{\mathrm{\ast 2}}$ above which punishers dominate (in panel (b)) for different values of $\theta$ as a function of initial percentage of punishers ${f}_{IP}$. With the increment of ${f}_{IP}$, the requirement of $r$ for punishers to establish monotonously and mildly decreases for any value of $\theta$. Notably, the ${r}^{\mathrm{\ast 1}}$ for smaller $\theta$ is significantly larger than that for larger $\theta$ for any initial percentage of punishers. This implies that higher tendency of migration (without punishing) does not provide punishers with higher opportunities to survive in direct competition with defectors. Although this is usually true for pure cooperators^34,37,38^, punishers need to be somewhat severe to gain a foot standing in the defectors, even when they are initially rare. However, when it comes to the case of punishers taking over defectors, whether larger $\theta$ is more advantageous is dependent on the initial fractions of punishers (see Fig. 3(b)). There exists a turning point around $0.04$, before which larger tendency to migrate favors punishers, and after which larger tendency to stay and punish is most profitable. Generally speaking, high migration tendency, albeit alleviating the high cost of punishing, dose not always offer punishers more chances of survival or domination. Except for the case that punishers are rare and struggling for domination, stay and punish is a better choice for punishers. In a word, punishers should be unflinching.

**Figure 4:**
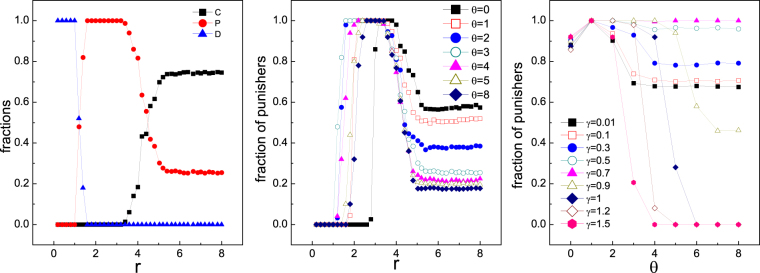
(**a**) Fractions of C, D, and P as functions of enhancement factor $r$. (**b**) Fraction of P as a function of $r$ for different values of $\theta$ when $\gamma =0.7$. (**c**) Fraction of P as a function of $\theta$ for different values of punishing cost $\gamma$ s when $r=3$. Population density is $\rho =0.5$.

So far we have discussed the impact of the choice between running and punishing on the evolution of punishers when they are confronted with defectors directly. However, as the second-order cooperators, besides the contribution made to the public pool as cooperators do, punishers also pay extra cost to regulate non-cooperators. Therefore it is unclear how defectors can survive when surrounded by cooperators. It is essential to investigate the mobility effect on punishers when cooperators are present, and to explore under what conditions mobility provides punishers with more privileges. Simulations on populations with initially equal percentage of cooperators and punishers show that stationary fractions of C, D, and P prevail at different intervals of enhancement factor $r$ respectively. For low $r$, population is occupied by only defectors. As $r$ increases, defectors are taken over by punishers. Further increasing $r$ brings prevailing cooperators and meanwhile punishers are suppressed but not eliminated (see Fig. 4(a)). The influence of mobility on defectors is two-fold with different $r$s: For small $r$ where only defectors and punishers coexist, there exists medium value of $\theta$ to ensure a easier domination of punishers (see Fig. 4(b)), which coincides with the observations in above case of merely competition between P and D in Fig. 1; For large $r$ where cooperators and punishers compete, high migration tendency $\theta =0$ is optimal for punishers, because large $r$ itself is sufficient to defeat defectors and less punishment avoids extra cost. Notably, for the former case that only P and D survive, punishing cost $\gamma$ also plays a role in determining the optimal value of $\theta$ which ensures highest P level (see Fig. 4(c)). However, always running ($\theta =0$) is never the best choice for any cost. If the cost is high, increment of $\theta$ significantly reduces punishers level. When cost is low, this effect is diminished. Large $\theta$ is detrimental for punishers’ triumph versus cooperators no matter the cost is large or small.

**Figure 5:**
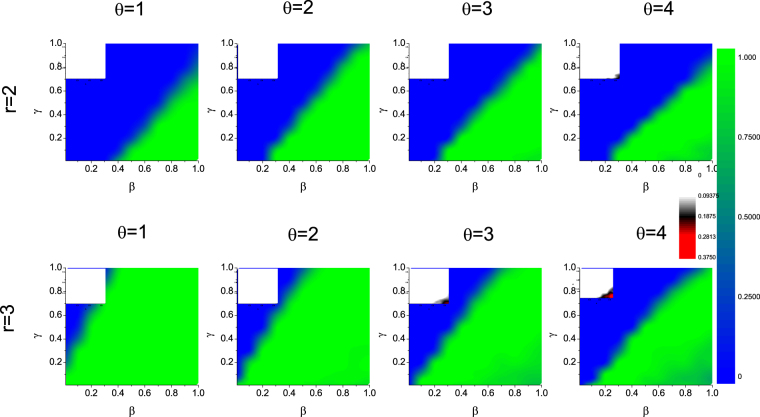
Fraction of punishers in dependence of punishing cost $\gamma$ and punishing intensity $\beta$ in the contour form when population are composed of C, D, and P. The first row corresponds to $r=2$ while the second row $r=3$. Different columns correspond to different thresholds to migrate $\theta =\mathrm{1,\; 2,\; 3,\; 4}$. The case for $\theta =0$ is not shown where defectors dominate for the whole given parameter area of $\gamma$ and $\beta$. Insects show the fraction of cooperators for the same parameter region as punishers.Initial fraction of C and P are both 0.25, and the rest D 0.5. Population density is $\rho =0.5$.

A detailed exploration of the punishment parameters’ influences on the punishers level (and cooperation level) when population is composed of initially equal percentage of cooperators and punishers for a variety of migration tendency is shown in Fig. 5. A common trait and straightforward observation for all these panels is that the right-bottom corner of the $\gamma -\beta$ plane, where punishment intensity is large but cost is low, is most favorable for defectors to be depressed and for the flourish of punishers. However, the area of this region’s reliance on $\theta$ shows different behaviors with varied $r$. For severe environment with relatively small $r$, champions in competition with defectors are mainly punishers. In this case, a medium $\theta$ value maximizes the area of inclusive cooperators (C and D). This is in accordance with the results observed above for direct competition between Cs and Ps in Fig. 2. While for a mild environment with larger $r$, cooperators gain more advantage by abstaining from paying the punishing cost, and take up quite a percentage in the final population. In this case, the area of punishment within the $\gamma -\beta$ plane shrinks with increasing $\theta$. In other words, it is better for punishers to run away in order to be advantageous when cooperators can survive in the defectors.

**Figure 6:**
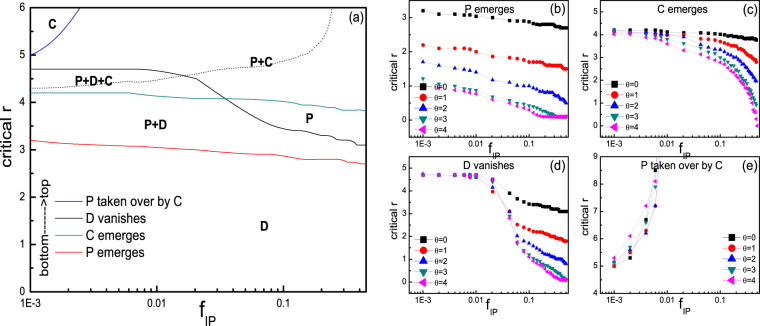
(**a**) Phase diagram for the stationary composition of population (C, D, and P) dependent on the initial fraction of punishers ${f}_{IP}$ and enhancement factor $r$ in the case of $\theta =0$. Legends indicates the crucial lines above which particular events occur as $r$ increases. Dashed line denote the $r$ values where equal percentage of Cs and Ps are obtained. As the initial fraction of punishers varies, the total percentage of initial cooperators and punishers remains 50%, with the remainder 50% being defectors. The influences of $\theta$ on each of the four lines shown in (**a**) are illustrated respectively in (**b**–**e**). For panels (**a–e**), the punishment cost is $\gamma =0.3$ and intensity is $\beta =1$. Population density is $\rho =0.5$.

Phase diagram for the stationary composition of population (C, D, and P) dependent on the initial fraction of punishers ${f}_{IP}$ and enhancement factor $r$ are shown in Fig. 6. Particularly, Fig. 6(a) shows the case for $\theta =0$. Lines in the figure indicate the critical values of $r$ where particular event occurs as a function of ${f}_{IP}$. Influences of the $\theta$ on each of these lines are respectively presented in Fig. 6(b–e). Notably, for very small ${f}_{IP}$, as $r$ increases, stationary composition of the population goes from full defectors to coexistence of P and D, coexistence of C, D, and P, coexistence of C and P, and finally pure cooperators when $r$ is sufficiently large. As ${f}_{IP}$ increases, requirement of $r$ for cooperators to dominate becomes so large that C usually coexists with P. Further increment of ${f}_{IP}$ leads to the domination of P over D before C emerges with larger $r$, and thus the coexistence of C, D, and P vanishes. Throughout the range of ${f}_{IP}$, larger $\theta$ reduces the critical $r$ for P to emerge in D (see Fig. 6(b)), indicating that lower tendency to migrate favors punishers. This is in line with the observations when only P and D exist initially in the system, since in such harsh environment C cannot survive and only by severe punishment of D rather than evasion can P persist. Meanwhile, larger $\theta$ also reduces the requirement of $r$ for C to emerge in stationary state (see Fig. 6(c)), indicating that larger tendency to punish reduces the relative advantage of P when accompanied by C. Lager $\theta$ more easily drives D to extinction (see Fig. 6(d)) especially when Ps have a larger proportion in the population, and also provides with punishers slightly larger region before the whole population is dominated by solely cooperators (see Fig. 6(e)). To sum up, larger tendency of running-away without punishing (smaller $\theta$) does not provide with punishers larger opportunities to survive either in the case of competing with defectors when $r$ is small, or significant privileges in the case of competing for domination with cooperators when $r$ is large, but only show its merits with medium $r$ by inhibiting the emergence of C when initially both C and P compete with defectors.

**Figure 7:**
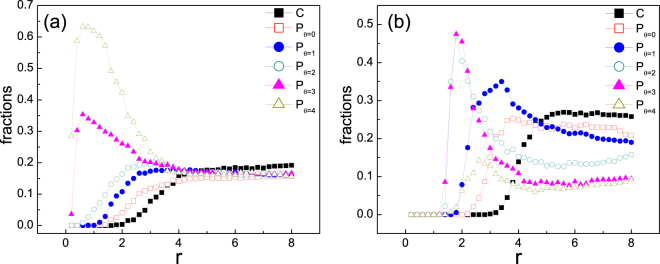
Fractions of different punisher classes and pure cooperators in the final state in dependence of enhancement factor $r$ when they start fighting with defectors simultaneously. Initially defectors take up 50% of the whole population and the rest are occupied by equal percentage of all these 6 types of individuals. Subscripts $\theta =\mathrm{0,\; 1...4}$ of P indicates the threshold of this type of P to migrate without punishing. Panel (a) corresponds to relatively low punishing cost $\gamma =0.3$ while Panel (b) high cost $\gamma =0.9$. Other parameters: $\beta =1$, and $\rho =0.5$. Data are obtained by averaging over 500 independent simulation runs.

Up to now, we have investigated the impact of different $\theta$ on the evolution of punishers solely. A natural issue arises that which of the $\theta$ will be favored by natural selection when these varied strategies fight with defectors simultaneously. Previous works have implied that the cooperation and competition among these types of individuals may lead to new findings^30,57^. To answer this question we have studied the evolution where initially equally percentage of Ps with varied $\theta$ together with pure cooperators (see Fig. 7). It can be found that for very large $r$, pure C is the ultimate winner, and none of these types of punishers outcompete cooperators in final fractions. However, the advantage of punishers arises when $r$ is not so large. And which P is most favored relies on the punishing parameters. For relatively low cost (see Fig. 7(a)), the type of punishers with lower tendency of migration (large $\theta$) is favorable. While for high cost (see Fig. 7(b)), punishers with a medium value of $\theta$ are most abundant in the population. These findings are quite similar to those observed above when punishers solely compete with cooperators and punishers.

## Discussion

Mobility is the essential feature of living organisms by which individuals leave unfavorable environment and increase the chances of survival. Mobility of individual plays significant role in answering the vital question of how cooperation is evolved when free-riding is apparently more beneficial. Recent advances have paid intensive attention to the role of punishing strategy. Yet as a higher-order cooperator, the emergence and persistence of punishers is still puzzling because punishers are easily taken over by the higher-order free-riders, i.e., the pure cooperators. In explaining the evolution of punishers, the mobility of punishers have long been ignored. To explore the effect of mobility on the survival of punishers, we have constructed a spatial model of public goods game played on square lattices with empty sites. Punishers are endowed with the capability of making the choice between staying still and punishing all defectors, and that of migrating to a neighboring empty site without punishing, with the decision dependent on the maximum number of defectors punishers can tolerate. Through Monte Carlo simulations, we have investigated the evolution of punishers affected by the migration tendency both in a scenario of punishers directly competing with defectors and in the case with cooperators, defectors, and punishers to check the condition under which migration offers more chances for punishers to survive, especially with the second-order free-riders, the cooperators. Furthermore, we have constructed a case where all these different types of punishers initially coexist in the evolution to check which one is most favored by natural selection.

Results indicate that the best $\theta$ relies on the cost of punishment. When punishers are faced with defectors directly, for considerably large cost, there exists medium value of $\theta$ to maximize its advantage, and medium $\theta$ also maximized the region of punishing parameters which ensures punishers’ domination. For cheap punishment with low cost, always punishing (large $\theta$) is best even if punishers are initially rare. When cooperators are also incorporated to check how mobility affect punishers’ chance of survival under the sabotage of second-order free-riders, it is found that $\theta$ plays different roles with varied environment harshness. In a harsh case with small $r$, cooperators cannot survive in the long run and thus results resemble the case above where punishers directly compete with defectors. While for the good environment where $r$ is large, Cs are more beneficial and small $\theta$ offers punishers more superiority if cost is large. For the low cost, the advantage of small $\theta$ is only observed when $r$ is medium and cooperators begin to coexist with defectors and punishers. Simultaneous competition and cooperation among different types of these punishers with varied $\theta$ also shows a reliance on the punishing cost. Heavier cost makes punishers with larger $\theta$ tend to be more inferior when $r$ is larger but shows its advantage with lower $r$.

The puzzling of punishment mainly lies in its cost relatively to cooperators, as well as being cooperators who become inferior when faced with defectors who contribute nothing. Hence previous works have proposed models incorporating more realistic traits by which the actual cost can be reduced when they are implemented^58,59^. One direction to explore is how the cost is born. Instances include those where cost are not only undertaken by the punishers, but also by those from higher hierarchies, the third party, or even cooperators^47,60^. Another way to consider is the punishment not always implemented, such as the coordination of punishment or the probabilistic execution^30,61^. Current model considers the migration to avoid the cost. Indeed it also resembles the probabilistic strategy, not designated uniformly but rather dependent on specific environment. Previous literature has reported that in the two-strategy social dilemmas the survival of cooperators is supported by the motion of influential players^33^. Similarly, we show that in the multi-player game of PGG with three strategies, overall cooperation can be enhanced if higher-order cooperators, the punishers, are allowed to move away from bad environment with certain probability. We hope this work contribute to further understanding of punisher’s survival in the evolutionary competition. And it helps to understand the role mobility plays when population are faced with various environments with differing levels of harshness.

## Methods

We consider a population of individuals playing public goods game staged on a $L\times L$ square lattice with periodic boundary conditions, where each site is either empty or occupied by one individual. Empty sites represent possible niches that individuals can migrate to^32,62^. The fraction of populated sites is denoted by the population density $\rho \in \mathrm{[0,1]}$. Each PGG is organized by the focal individual resided on one site, and participants include the individuals located in the direct neighboring sites (of number $k=8$, the Moore neighborhood). Initially each player is designated as one of the three types: cooperator (C), defector (D), and punisher (P).

In the evolution each elementary step consists of the following two stages. Firstly, a PGG is held. Each cooperator or punisher contributes to the common pool at a cost $c$, while defectors do nothing. Without loss of generality, we set $c=1$ throughout this work. Then all the contributions are summed up and multiplied by an enhancement factor $r$, and are then equally distributed among all the group members, despite their contributions. Secondly, recognizing the number of defectors in the group, each punisher makes a decision to punish all the defectors or to leave current group. If the number of defectors is no more than the threshold $\theta$, punisher will punish each defector in this group by reducing its payoff by $\beta$ at a cost of $\gamma$ as commonly assumed in previous literatures^27,28^. Otherwise, the punisher leaves current site and migrates to a randomly chosen empty sites in the neighborhood. Thus magnitude of $\theta$ reflects one’s punishment tendency, or inverse migration tendency. Each evolutionary generation is composed of many elementary steps such that on average each individual has organized once the PGG. After that, each individual $i$ updates its strategy synchronously by copying the strategy of a randomly chosen neighbor $j$ with an imitation probability^63^ given by

1 $$P(i\leftarrow j)=\frac{1}{1+\exp [({P}_{i}-{P}_{j})/\kappa ]}$$

where $\kappa$ measures the magnitude of noise to permit irrational choices. Smaller value implies that superior strategies are more favored to be adopted, albeit it is not impossible to employ the suboptimal strategies. In the present work we have designated $\kappa =0.1$ which depicts a scenario of strong selection where beneficial strategies are more likely to survive under evolution pressure.

During one full Monte Carlo step (MCS), all individuals in the population receive a chance once on average to adopt another strategy. To avoid the frozen state in which some individual clusters are isolated and thus have no chance of interaction with the rest of the population, a small probability $\varepsilon =0.01$ is applied for any individual to migrate randomly to an empty neighboring sites. And we have checked that the influence of this mobility on the final evolution state is negligible. The population density $\rho$ may affect the evolution of strategies on networks with empty sites as previously reported^38,62^, yet in current work we mainly focus on the mobility of punishers and assume a fixed population density $\rho =0.5$ throughout the paper. Depending on the proximity to phase transition points and the typical size of emerging spatial patterns, the linear system size was varied from *L* = 100 to 400 and the relaxation time was varied from ${10}^{4}$ to ${10}^{6}$ MCS to ensure proper statistical accuracy. The reported fractions of competing strategies were determined in the stationary state when their average values became time-independent. Alternatively, we have averaged the outcomes over 50 to 2000 independent runs when the system terminated into a uniform absorbing state.

### Data availability

The authors declare that all data supporting the findings of this study are included in this published article.

## References

[1] Axelrod R, Hamilton WD (1981). The evolution of cooperation. Science.

[2] Henrich, J. & Henrich, N. *Why Humans Cooperate: A Cultural and Evolutionary Explanation* (Oxford University Press, Oxford, 2007).

[3] Bowles, S. & Gintis, H. *A Cooperative Species: Human Reciprocity and Its Evolution* (Princeton University Press, Princeton, NJ, 2011).

[4] Rand DG, Nowak MA (2013). Human cooperation. Trends Cog. Sci.

[5] Pennisi E (2005). How did cooperative behavior evolve?. Science.

[6] Colman AM (2006). The puzzle of cooperation. Nature.

[7] Nowak MA (2006). Five rules for the evolution of cooperation. Science.

[8] Sigmund, K. *The Calculus of Selfishness* (Princeton University Press, Princeton, NJ, 2010).

[9] Poteete, A. R., Janssen, M. A. & Ostrom, E. *Working Together: Collective Action, the Commons, and Multiple Methods in Practice* (Princeton University Press, Princeton, 2010).

[10] Wang X, Perc M, Liu Y, Chen X, Wang L (2012). Beyond pairwise strategy updating in the prisoner’s dilemma game. Sci. Rep..

[11] Yamagishi T (1986). The provision of a sanctioning system as a public good. J. Pers. Soc. Psychol..

[12] Ostrom E, Walker J, Gardner R (1992). Covenants with and without a sword: Self-governance is possible. Am. Polit. Sci. Rev..

[13] Sigmund K, Hauert C, Nowak MA (2001). Reward and punishment. Proc. Natl. Acad. Sci. USA.

[14] Fehr E, Gachter S (2002). Altruistic punishment in humans. Nature.

[15] Sigmund K (2007). Punish or perish? Retaliation and collaboration among humans. Trends Ecol. Evol..

[16] Raihani NJ, Thornton A, Bshary R (2012). Punishment and cooperation in nature. Trends Ecol. Evol..

[17] Perc M, Szolnoki A (2015). A double-edged sword: Benefits and pitfalls of heterogeneous punishment in evolutionary inspection games. Sci. Rep..

[18] Cong R, Li K, Wang L, Zhao Q (2016). Cooperation induced by wise incentive allocation in spontaneous institution. EPL.

[19] Boyd R, Gintis H, Bowles S, Richerson PJ (2003). The evolution of altruistic punishment. Proc. Natl. Acad. Sci. USA.

[20] Helbing D, Szolnoki A, Perc M, Szabó G (2010). Evolutionary establishment of moral and double moral standards through spatial interactions. PLoS Comput. Biol..

[21] Szolnoki A, Szabó G, Perc M (2011). Phase diagrams for the spatial public goods game with pool punishment. Phys. Rev. E.

[22] Szolnoki A, Szabó G, Czakó L (2011). Competition of individual and institutional punishments in spatial public goods games. Phys. Rev. E.

[23] Perc M, Szolnoki A (2012). Self-organization of punishment in structured populations. New J. Phys..

[24] Wang W-X, Lai Y-C, Grebogi C (2016). Data based identification and prediction of nonlinear and complex dynamical systems. Phys. Rep..

[25] Han X (2017). Emergence of communities and diversity in social networks. Proc. Natl. Acad. Sci. USA.

[26] Fowler JH (2005). Altruistic punishment and the origin of cooperation. Proc. Natl. Acad. Sci. USA.

[27] Hauert C, Traulsen A, Brandt H, Nowak MA, Sigmund K (2007). Via freedom to coercion: The emergence of costly punishment. Science.

[28] Sigmund K, De Silva H, Traulsen A, Hauert C (2010). Social learning promotes institutions for governing the commons. Nature.

[29] Boyd R, Gintis H, Bowles S (2010). Coordinated punishment of defectors sustains cooperation and can proliferate when rare. Science.

[30] Chen X, Szolnoki A, Perc MCV (2015). Competition and cooperation among different punishing strategies in the spatial public goods game. Phys. Rev. E.

[31] Majeski S, Linden G, Linden C, Spitzer A (1999). Agent mobility and the evolution of cooperative communities. Complexity.

[32] Vainstein MH, Silva ATC, Arenzon JJ (2007). Does mobility decrease cooperation?. J. Theor. Bio..

[33] Droz M, Szwabiński J, Szabó G (2009). Motion of influential players can support cooperation in prisoner’s dile. mma. Eur. Phys. J. B.

[34] Helbing D, Yu W (2009). The outbreak of cooperation among success-driven individuals under noisy conditions. Proc. Natl. Acad. Sci. USA.

[35] Wu T, Fu F, Wang L (2011). Moving away from nasty encounters enhances cooperation in ecological prisoner’s dilemma gam. e. PLoS ONE.

[36] Szolnoki A, Perc M (2013). Correlation of positive and negative reciprocity fails to confer an evolutionary advantage: Phase transitions to elementary strategies. Phys. Rev..

[37] Vainstein MH, Brito C, Arenzon JJ (2014). Percolation and cooperation with mobile agents: Geometric and strategy clusters. Phys. Rev. E.

[38] Cong R, Wu B, Qiu Y, Wang L (2012). Evolution of cooperation driven by reputation-based migration. PLoS ONE.

[39] Szolnoki A (2014). Cyclic dominance in evolutionary games: a review. J. R. Soc. Interface.

[40] Wang X, Chen X, Wang L (2015). Evolutionary dynamics of fairness on graphs with migration. J. Theor. Biol..

[41] Zhang Y, Su Q, Sun C (2016). Intermediate-range migration furnishes a narrow margin of efficiency in the two-strategy competition. PLOS ONE.

[42] Zhang Y, Liu A, Sun C (2016). Impact of migration on the multi-strategy selection in finite group-structured populations. Sci. Rep..

[43] Sicardi EA, Fort H, Vainstein MH, Arenzon JJ (2009). Random mobility and spatial structure often enhance cooperation. J. Theor. Biol..

[44] Zhang Y, Fu F, Chen X, Xie G, Wang L (2015). Cooperation in group-structured populations with two layers of interactions. Sci. Rep..

[45] Wu T, Fu F, Zhang Y, Wang L (2012). Expectation-driven migration promotes cooperation by group interactions. Phys. Rev. E.

[46] Yang H-X, Wu Z-X, Wang B-H (2010). Role of aspiration-induced migration in cooperation. Phys. Rev. E.

[47] Chen X, Szolnoki A, Perc M (2014). Probabilistic sharing solves the problem of costly punishment. New J. Phys..

[48] Bull J, Rice W (1991). Distinguishing mechanisms for the evolution of co-operation. J. Theor. Biol..

[49] Baumard N, André J-B, Sperber D (2013). A mutualistic approach to morality: The evolution of fairness by partner choice. Behav. Brain Sci..

[50] Wu T, Fu F, Zhang Y, Wang L (2013). Adaptive role switching promotes fairness in networked ultimatum game. Sci. Rep..

[51] Szabó G, Fáth G (2007). Evolutionary games on graphs. Phys. Rep..

[52] Droz M, Pȩ kalski A (2016). A. On the role of fluctuations in the modeling of complex syste. ms. Front. Phys..

[53] Chen X, Fu F, Wang L (2008). Influence of different initial distributions on robust cooperation in scale-free networks: A comparative study. Phys. Lett. A.

[54] Lei C, Jia J-Y, Chen X-J, Cong R, Wang L (2009). Prisoner’s dilemma game on clustered scale-free networks under different initial distributions. Chin. Phys. Lett.

[55] Wang X, Chen X, Wang L (2014). Random allocation of pies promotes the evolution of fairness in the ultimatum game. Sci. Rep..

[56] Szolnoki A, Perc M (2016). Leaders should not be conformists in evolutionary social dilemmas. Sci. Rep..

[57] Szolnoki A, Perc M (2016). Competition of tolerant strategies in the spatial public goods game. New J. Phys..

[58] Chen X, Sasaki T, Perc M (2015). Evolution of public cooperation in a monitored society with implicated punishment and within-group enforcement. Sci. Rep..

[59] Li K, Cong R, Wu T, Wang L (2015). Social exclusion in finite populations. Phys. Rev. E.

[60] Zhang C, Zhu Y, Chen Z, Zhang J (2017). Punishment in the form of shared cost promotes altruism in the cooperative dilemma games. J. Theor. Biol..

[61] Szolnoki A, Perc M (2013). Effectiveness of conditional punishment for the evolution of public cooperation. J. Theor. Biol..

[62] Vainstein MH, Arenzon JJ (2001). Disordered environments in spatial games. Phys. Rev. E.

[63] Szabó G, Tőke C (1998). Evolutionary prisoner’s dilemma game on a square lattice. Phys. Rev. E.

